# Commissioning and implementation of a stereotactic conformal radiotherapy technique using a general‐purpose planning system

**DOI:** 10.1120/jacmp.v5i3.1948

**Published:** 2004-10-21

**Authors:** M. Amin Mosleh‐Shirazi, Vibeke N. Hansen, Peter J. Childs, Alan P. Warrington, Frank H. Saran

**Affiliations:** ^1^ Joint Department of Physics The Institute of Cancer Research and The Royal Marsden NHS Trust Sutton Surrey SM2 5PT United Kingdom; ^2^ Department of Radiotherapy The Institute of Cancer Research and The Royal Marsden NHS Trust Sutton Surrey SM2 5PT United Kingdom

**Keywords:** pediatric stereotactic conformal radiation therapy, treatment‐planning system commissioning, conformal block production, CT inhomogeneity correction, treatment isocenter verification

## Abstract

The purpose of this paper is to report on commissioning and clinical implementation of a customized system for pediatric stereotactic conformal radiotherapy (SCRT). The system is based on the Pinnacle treatment‐planning system and its interfaces with other equipment: (1) Beam models were optimized for our compact blocking system and a new LINAC. (2) Three CT‐to‐density conversion tables were evaluated, one using tabulated data for a commercial phantom, the second including additional points from the manufacturer's data for the inserts in an in‐house phantom, and the third using measured densities for the in‐house phantom materials combined with tabulated data for the commercial phantom. (3) Blocks were transferred to a computerized block cutter using in‐house software that extracted the block shape from the export file and custom‐fitted the additional necessary shapes. (4) In the absence of a DICOM RT Image link, a method based on screen data capture was used to export digitally reconstructed radiographs (DRRs) to two portal imaging systems for treatment verification. Lens shielding by multileaf collimation in the anterior‐posterior isocenter verification field was investigated. (1) Computed dose distributions using the beam models agreed with measurements well within published acceptability criteria. A difference of up to 1.0 mm was measured between the beam's eye views of aperture blocks and computed 50% isodose contours for a 2×2×2 mm dose calculation grid. (2) The third table, which included measured densities, improved the accuracy of the calculated isocenter dose by up to 0.5% in typical patient SCRT treatments and up to 1.0% in a phantom with 5‐cm diameter inhomogeneity inserts. (3) The block export and customization process was shown to introduce no additional uncertainty. A 1‐mm block production uncertainty was measured using film dosimetry on six blocks. (4) The DRR transfer method did not introduce uncertainty into the process. Verification field shielding reduced lens dose by 12 to 15 times. In conclusion, this customized system for planning and verification of pediatric SCRT provides a high level of precision as well as reasonable practical efficiency.

PACS numbers: 87.53.Kn, 87.53.Ly, 87.53.Oq, 87.53.Tf, 87.53.Uv

## I. INTRODUCTION

Small‐to‐medium sized benign or low‐grade childhood brain tumors are routinely treated at The Royal Marsden Hospital with fractionated stereotactic conformal radiotherapy (SCRT) using static noncoplanar conformal fields. The patient is immobilized in either an in‐house relocatable stereotactic frame designed for children or a high‐precision thermoplastic cast system.^(1)^ Gross tumor volume delineation is performed based on coregistered CT‐MR images.

Customized blocks offer the highest conformity to targets. We use an in‐house, compact blocking system for SCRT. Its compactness offers a greater beam access compared to tray‐mounted blocks or tertiary multileaf collimators (MLCs), thereby reducing the overlap of noncoplanar beam paths. The positional reproducibility of the SCRT blocks is better than 0.5 mm,^(1)^ and the beam passes through the block aperture unattenuated since no accessory tray is used.

The Pinnacle 3D treatment‐planning system (ADAC Laboratories, Philips Medical Systems, Milpitas CA) was previously commissioned for general treatment planning at our center.^(2)^ As with most other standard commissioning processes for 3D conformal radiotherapy (RT), the commissioned aspects had included beam modeling for the required field size range (3 cm to 40 cm), density heterogeneity correction, oblique and asymmetric field dosimetry, and irregular field shaping using MLCs and standard blocks, as well as quality assurance testing of dose volume histograms and digitally reconstructed radiographs (DRRs). We specify our SCRT fields in Pinnacle as static beams since the option for stereotactic arcs with circular collimators does not reflect our treatment setup. The SCRT technique uses specialized hardware as well as further planning system functionality than routine planning. The special requirements of SCRT necessitated a customized system of planning and verification for this technique. This paper reports the previously unpublished aspects of the commissioning process for this SCRT system and describes its clinical implementation at our center. In addition, following the previous publication,^(2)^ further investigations have been carried out on Pinnacle; we have incorporated them into SCRT planning and report on them in this paper.

Pinnacle forms the central component of the SCRT system. The other components are CT and MRI scanners, the stereotactic frame or high‐precision cast, our SCRT blocks, a computerized block cutter, and electronic portal imaging (EPI) systems. CT‐MR image registration is not covered in this paper. Our patient immobilization techniques were previously reported,^(1)^ and a separate small‐field beam model had been developed for SCRT, as previously described.^(2)^ The requirements for the rest of the system are introduced below and addressed in this paper, starting from dose calculation, then block production, and finally treatment verification:

1. Another SCRT beam model for a different LINAC, optimized for our SCRT blocks and a limited field size range, as with the first model.
2. Determination of the relationship between Pinnacle's 50% isodose contour, beam's eye view (BEV) representation of an aperture block, and its projection to the plane of the isocenter.
3. A practical method to produce a more representative CT‐to‐density conversion table, compared with that achieved by adoption of manufacturers’ data for density inhomogeneities in calibration phantoms.
4. Export, editing, and introduction of additional shapes (central locating hole and outer fixing extensions) needed for pouring lead alloy and fixation with our SCRT blocks, and the subsequent import of the resulting block shapes into a computerized hot‐wire block cutter.
5. Measurement of the accuracy of our block production technique.
6. An interim solution to the problem of importing DRRs into the EPI systems as reference images for treatment verification, in the absence of a DICOM RT Image link for DRR export from Pinnacle.
7. Reduction of lens dose from isocenter verification fields for children.

The small‐field aspect and the greater beam modeling accuracy needed for SCRT add to most of the above requirements to make this commissioning process nonstandard. Since the SCRT blocks are in‐house and only used for this application, steps 1, 2, 4, and 5 are specific to this customized system. In addition, step 7 may be deemed necessary for children only and is therefore specific to such patients. Step 3 may only be carried out to the extent reported here if the required level of accuracy merits it, while step 6 is only required where DRR‐based verification is performed using exported (not printed‐out) images and if a DICOM RT Image link for DRRs is not available.

## II. MATERIALS AND METHODS

### A. Beam modeling

Machine data incorporating small‐field beam models, manually optimized for our SCRT blocks, had been created on Pinnacle for the 6‐MV beam of an Elekta SL15 LINAC (Elekta AB, Stockholm, Sweden).^(2)^ Instead of using the published or interpolated spectra available on the system, a Monte Carlo–generated photon energy spectrum had been used in that model.^(2)^ The commissioning process was repeated for the 6‐MV beam of an Elekta Precise LINAC. To create beam models for general planning with this LINAC, the spectrum and the other beam parameters had been auto‐modeled on Pinnacle and subsequently adjusted manually for improved matching to measurements, a single model representing open fields for the 3 cm to 40 cm range of field sizes and another one for wedged fields. For SCRT, the general‐planning models were used as the starting point for optimization in the field size range 3 cm to 10 cm but recomputed with a 1.5‐mm fluence grid resolution instead of 2.5 mm. Given the reduced field size range, the models could be further optimized for unblocked beams to improve agreement with measurements. Based on comparisons of computed and measured planar dose distributions and dose profiles for blocked fields, no further changes were necessary for the open fields, while the wedge model required manual adjustments. The main changes to the wedge model were regarding the incident fluence parameters: To increase the magnitude of off‐axis profiles, *incident fluence increase per unit distance and incident fluence cone radius* were changed from 0.004/cm and 27.6 cm to 0.007/cm and 2.5 cm, respectively. In addition, *source size* perpendicular to the gantry axis was decreased from 0.13 cm to 0.03 cm in order to sharpen the penumbra along that direction. The acceptability criteria applied to SCRT blocked fields included a 1% and 0.5‐mm tolerance for dose profiles and a 1.0‐mm tolerance for isodoses in planar dose distributions, which were tighter than the published criteria^(^ ^3^ ^–^ ^5^ ^)^ that had been used for the general‐planning beam models.

Pinnacle versions 5.2g to 6.2b were used. The most accurate dose calculation algorithm in Pinnacle (and other planning systems that employ an analytical approach) is collapsed‐cone convolution‐superposition, which was the algorithm selected during commissioning and clinical use. During model optimization, a 1.5×1.5×1.5 mm dose calculation grid was used, which was the finest resolution possible with the available system memory at the time. In routine clinical SCRT planning, a clinically accepTable 2.0×2.0×2.0 mm grid is selected to reduce dose calculation time.

The measurement devices used for comparison with calculations were 0.13 cm^3^ and 0.6 cm^3^ ionization chambers, photon diode, and film. The beam penumbra measurements presented in this paper were carried out with Kodak X‐Omat V films at 5‐cm and 10‐cm depths in water‐equivalent plastic for a 90‐cm source‐to‐surface distance (SSD). Calibration irradiations were made with films orthogonal to the beam axis (to match the measurement conditions) and processed together with the measurement films, producing a 13‐point optical‐density‐to‐dose conversion curve. The films were scanned with 0.2‐mm resolution in a Vidar VXR‐12 scanner (Vidar Systems Corporation, Herndon VA) and analyzed using the Wellhöfer WP700 software (Scanditronix Wellöfer GmbH, Schwarzenbruck, Germany).

### B. CT‐to‐density table

Following the commissioning of a new CT scanner for RT planning, an accurate CT‐to‐density conversion table specific to the scanner was required to obtain correct dose calculations based on voxel‐by‐voxel inhomogeneity correction. The conversion table in Pinnacle is in the form of CT number (numerically equal to Hounsfield units plus 1000) versus physical (mass) density. Density values are used in Pinnacle through linear interpolation from the nearest data points in the conversion table in order to (1) look up mass attenuation coefficients and (2) scale the dose deposition kernel to include the effects of inhomogeneities on scattered radiation. A common method of acquiring the conversion data from CT intensity values to density information is by scanning a calibration phantom. In addition to the RMI Electron Density Phantom model 465^(6)^ (Gammex RMI, Middleton WI) containing several inserts of different inhomogeneities, we used an in‐house CT quality assurance (QA) phantom to provide an independent check of the RMI phantom data as well as additional data points.

The CT QA phantom is cylindrical, water‐filled, and 25 cm in diameter. It contains six cylindrical inhomogeneity inserts, each 5 cm in diameter, isotropically arranged at 60° intervals with their centers 8 cm away from the phantom center. A 9‐cm diameter Lucite cylinder forms the central portion of the phantom. The phantom and the inserts are 16 cm and 15 cm long, respectively. The inserts contain water and air as well as the following tissue‐equivalent materials (manufactured at St. Bartholomew's Hospital, London, U.K.): lung (LN10), average bone (RB2), and cortical bone (SB3). The physical densities of the tissue substitutes in the phantom were determined from accurate measurements of insert dimensions and weight, repeated with different equipment and checked against published or manufacturer's data. The uncertainty associated with these measurements resulted in a <0.5% error in the density values.

A cavity for fitting a 0.6‐cm^3^ ionization chamber at the center of the phantom allowed dose measurements with beams passing through each insert. With the isocenter at the center of the phantom, the central axis of a beam through the center of an insert traversed approximately 2 cm of water, 5 cm of the inhomogeneity, and 5 cm of Lucite before reaching the chamber.

**Figure 1:**
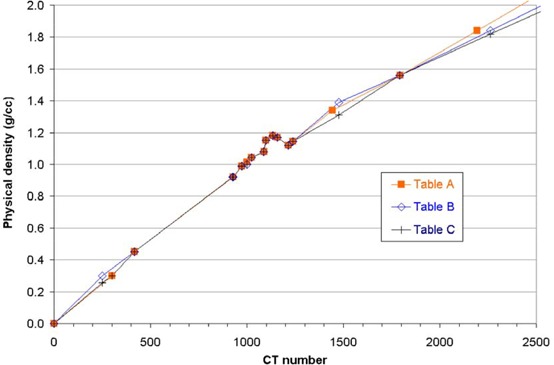
The three CT‐to‐density conversion tables studied. The data points are joined by straight lines and linearly extrapolated beyond the highest value in all three cases.

The corresponding setup was replicated in Pinnacle based on a helical CT scan of the phantom taken using a GE HiSpeed QX/i CT scanner (General Electric Company, Fairfield CT) operated at 120 kVp. Figure 1 shows the three CT‐to‐density conversion tables studied here. Table A was created using only the physical density data for inhomogeneity inserts in the RMI phantom as given in the manufacturer's manual and reported by Constantinou et al.^(6)^ plus a data point for the origin (0, 0). The CT number for each insert can be dependent on the scanner and scanning parameters; therefore, the CT numbers were obtained from the reconstructed data set by averaging pixel values in regions of interest created on the inserts in Pinnacle (as opposed to using the corresponding tabulated CT numbers). To study the impact of using data points from additional phantoms, this conversion table was slightly modified to create Table B; density values for the lung and two bone inserts in the CT QA phantom (based on the manufacturer's data^(^ ^7^ ^,^ ^8^ ^)^) were added, and the closest data points were removed from the original table. A density of $0.30\;{g/\text{cm}}^{3}$ was used for the LN10 lung‐equivalent material since the only available data were given as a range (0.25 to $0.35\;{g/\text{cm}}^{3}$). A data point for water (1000, 1) was also added. To investigate the impact of using measured density values, the manufacturer's data for the CT QA phantom inserts were then replaced by our measured densities, and a lung‐equivalent data point from the RMI phantom was reincluded to produce Table C. Dose calculations at the center of the in‐house phantom were compared with measurements for the three conversion tables. The computed doses at the isocenter based on the three tables were also compared for standard SCRT beams on a typical patient's CT data set.

### C. Block export, customization, and production

A HEK computerized hot‐wire block cutter (HEK Medizintechnik GmbH, Lübeck, Germany) was used for block production. A Microsoft Windows version of the HEK software (Autimo 2D v2.1) with a Pinnacle interface was commissioned. Software was written to carry out automatic shape modification based on the aperture and rectangular outer block edges exported from Pinnacle and to create files for import into the HEK system.

The overall block production accuracy was measured for six SCRT blocks using calibrated films at 10 cm depth in water‐equivalent plastic with a 90‐cm SSD.

### D. Treatment verification

The DRR transfer method from Pinnacle to the EPI systems is based on screen data capture and a subsequent use of file transfer protocol (FTP) communication. The DRRs transferred include the treatment beams as well as isocenter verification fields. The latter are an orthogonal pair of anterior‐posterior (AP) and lateral beams with the same isocenter as the treatment beams, the sizes of which are adjusted to visualize extensive bony anatomy (to be used for setup verification) while minimizing the dose to critical structures. The EPI systems used were Theraview (Cablon Medical BV, Leusden, The Netherlands), which is a camera‐based imager with a metal‐phosphor detector, and iViewGT (Elekta AB, Stockholm, Sweden), incorporating an amorphous silicon detector. Simple geometric test patterns showing the central axis and several off‐axis points were designed on Pinnacle and transferred to the EPI systems to check the fidelity of the export process. A method of isocenter verification for children, in which MLC leaves were used in the AP field to shield the lenses, was designed and evaluated in order to reduce the additional exposure from verification beams.

## III. RESULTS

### A. Beam modeling

**Figure 2:**
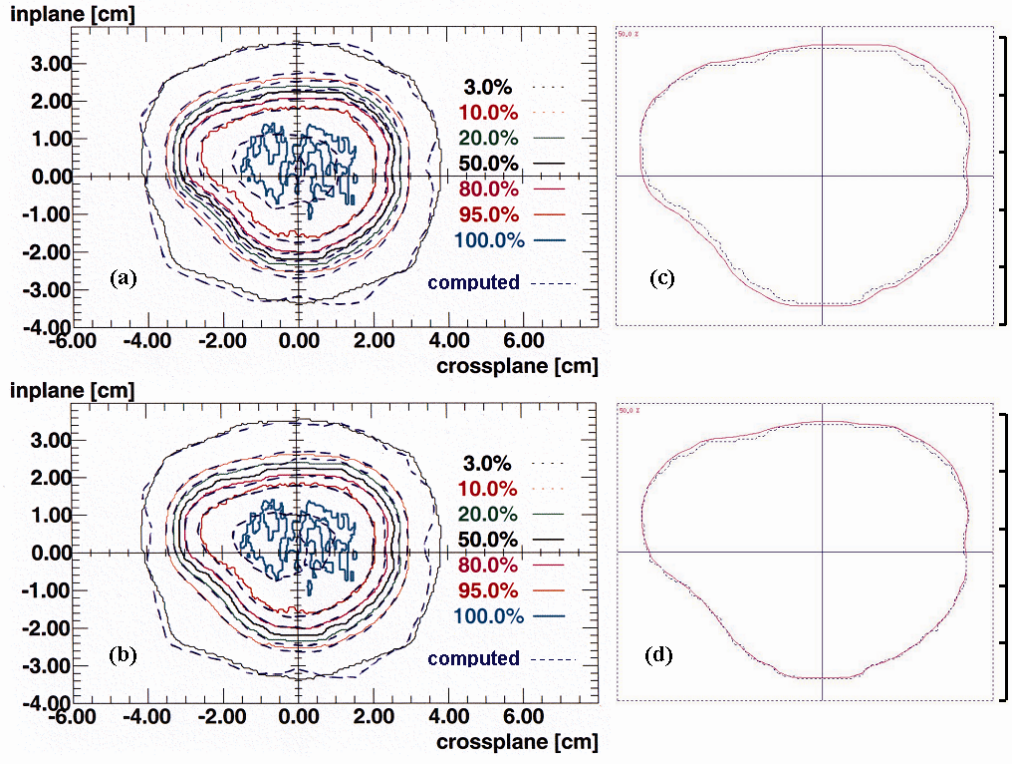
(a) Computed (dashed blue lines) and measured planar dose distributions at 10 cm depth from a block aperture created within Pinnacle and made from lead alloy. (b) The corresponding computed distribution for a block digitized from the 50% isodose from the above film and its comparison with the measurement. (c) and (d) The computed 50% isodose line at 10 cm depth (purple) versus the block shape projected to the 10 cm deep plane by Pinnacle (blue) for the blocks in (a) and (b), respectively.

The original single, open‐field, general‐planning model used for field sizes 3 cm to 40 cm was found to also satisfy the SCRT acceptability criteria for 3‐cm to 10‐cm blocked fields. The wedge model, however, required further manual optimization. The degree of agreement between calculations and measurements for the Elekta Precise LINAC was very similar (within 0.5% and 0.5 mm) to the previously reported Elekta SL15 model^(2)^ for central‐axis percentage depth doses and dose distributions at 5‐cm and 10‐cm depths. Therefore, detailed comparison results will not be repeated here. As a typical example, Fig. 2(a) shows a comparison of film measurement with a planar dose distribution (at 10‐cm depth) from a block aperture created within Pinnacle to fit a patient's planning target volume (PTV) and computed with a 2×2×2 mm dose grid. Both distributions were normalized on the central axis at 10‐cm depth. The measured isodose lines, which include block production errors, mostly agree to within 1.0 mm of the modeled distribution, maximum discrepancy being 1.2 mm. The uncertainties due to block production were then removed by digitizing the block shape into Pinnacle from the 50% isodose contour on the irradiated film (Fig. 2(b). A comparison of the digitized and original contours showed that the discrepancy introduced by digitization was negligible (<0.3 mm). Having matched the 50% contours, the 95%, 80%, 20%, and 10% isodoses agree in most places to within 0.5 mm, maximum difference being 1.0 mm. The 3% isodose, 1 cm outside the block aperture, shows a reasonable discrepancy of less than 2 mm in most places and a maximum difference of 4 mm (such differences in this relatively shallow dose‐gradient region represent very small dose errors).

Measured 80% to 20% and 95% to 50% penumbra for our SCRT blocks were 3 mm to 4 mm and 3 mm to 5 mm, respectively. An automatically produced 4‐mm block margin around the PTV in clinical plans results in sufficient coverage by the 95% isodose.

Comparisons of the beam shape and the computed 50% isodose line (both produced by Pinnacle at a 10‐cm deep plane) are shown in Figs. 2(c) and 2(d) for the above two cases. A difference of up to 1.0 mm exists between the block edges and the 50% isodose. A slightly better match is shown by the digitized block compared with the original automatically generated one despite identical dose calculation methods and matrices and the same isocenter and field jaw positions. With a 1×1×1 mm dose calculation grid, however, the maximum mismatch of the block border with the 50% isodose was reduced to 0.7 mm for both the original and the digitized block. The projection of beam shapes to the 10‐cm deep plane agreed with the corresponding shapes in the BEV window to within 0.5 mm, demonstrating that it is valid to use the projection to represent a beam from Pinnacle for comparisons with measurements.

### B. CT‐to‐density table

The density of the LN10 lung‐equivalent material in the CT QA phantom was measured to be $0.256\pm 0.001\;{g/\text{cm}}^{3}$. This is outside the range 0.288 to $0.521\;{g/\text{cm}}^{3}$ measured by the manufacturer on a series of material subtypes^(9)^ but is within the 0.25 to $0.35\;{g/\text{cm}}^{3}$ range given for commercial samples, the variation being due to differences in the manufacturing process.^(8)^ The measured density of the RB2 average‐bone substitute was $1.312\pm 0.007\;{g/\text{cm}}^{3}$, which was 6% lower than the manufacturer's data $\left(1.39\;{g/\text{cm}}^{3}\right)$.^(8)^ The density of the SB3 cortical‐bone‐equivalent material was measured to be $1.819\pm 0.009\;{g/\text{cm}}^{3}$, lower than the reported $1.84\;{g/\text{cm}}^{3}$ by 1%.^(^ ^6^ ^–^ ^8^ ^,^ ^10^ ^)^

**Figure 3:**
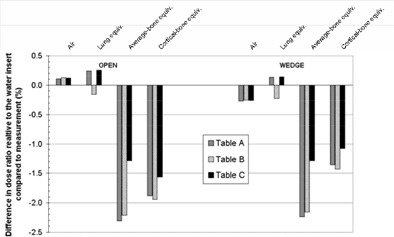
Comparison of the isocenter dose computed based on the three CT‐to‐density conversion tables for 5×5 cm beams through different inhomogeneity inserts in the in‐house phantom. The differences between calculated and measured doses for each inhomogeneity are plotted as percentages of the isocenter dose for the water insert.

In Fig. 3, relative dose calculations using the three CT‐to‐density tables are compared with measurements at the center of the CT QA phantom (isocenter) for 5×5 cm open and wedged 6‐MV beams of an Elekta Precise LINAC. Each plotted point is the calculated‐minus‐measured percentage difference between the ratios of the isocenter dose for a beam through the inhomogeneity insert relative to the isocenter dose for the water insert. Plotting doses relative to water highlights the differences due to inhomogeneities. The agreement between measurements and computed doses with the three tables is within 0.02% and 0.4% for the air and lung‐equivalent inhomogeneities, respectively. For both bone‐equivalent materials, the computed doses are lower than measured for the three tables, Table C reducing the underestimate (averaged over both bone inhomogeneities and the open and wedged beams) from about 2.0% of the isocenter dose to 1.3%, compared with Tables A and B. The greatest improvement is for average bone (1.0% of isocenter dose for both open and wedged beams). Similar results were obtained with the SCRT beam model of the SL15 LINAC.

As expected, the observed differences between the conversion tables were reduced when SCRT beams were computed on a typical CT data set of a nine‐year‐old patient. For most beams passing through the cranium, the differences in the dose to the isocenter were negligible (0.1% to 0.2%), with the dose to some off‐axis points being up to 0.5% different.

Posterior‐inferior‐oblique beams through the region of temporal and sphenoid bones aimed at small targets at the base of skull showed the greatest difference in isocenter dose, Table C predicting up to 0.5% higher dose than Table A (due to a change in the derived effective radiological depth of up to 2 mm).

**Figure 4:**
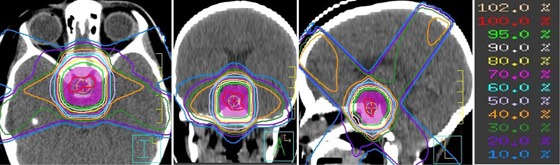
Transverse, coronal, and sagittal dose distributions resulting from the standard four‐field SCRT beam geometry, computed using the small‐field beam model.

Example dose distributions computed using a clinical beam model for our standard SCRT plan are shown in Fig. 4. There are four noncoplanar oblique beams from the anterior‐superior, posterior‐superior, right‐posterior‐inferior and left‐posterior‐inferior directions. Isodoses ≥50% closely conform to the shape of the PTV (purple shaded region). It has been shown that target coverage and normal brain sparing are usually not improved by using more than 4 to 6 beams to treat the sellar and parasellar tumors considered for SCRT.^(11)^

### C. Block export, customization, and production

**Figure 5:**
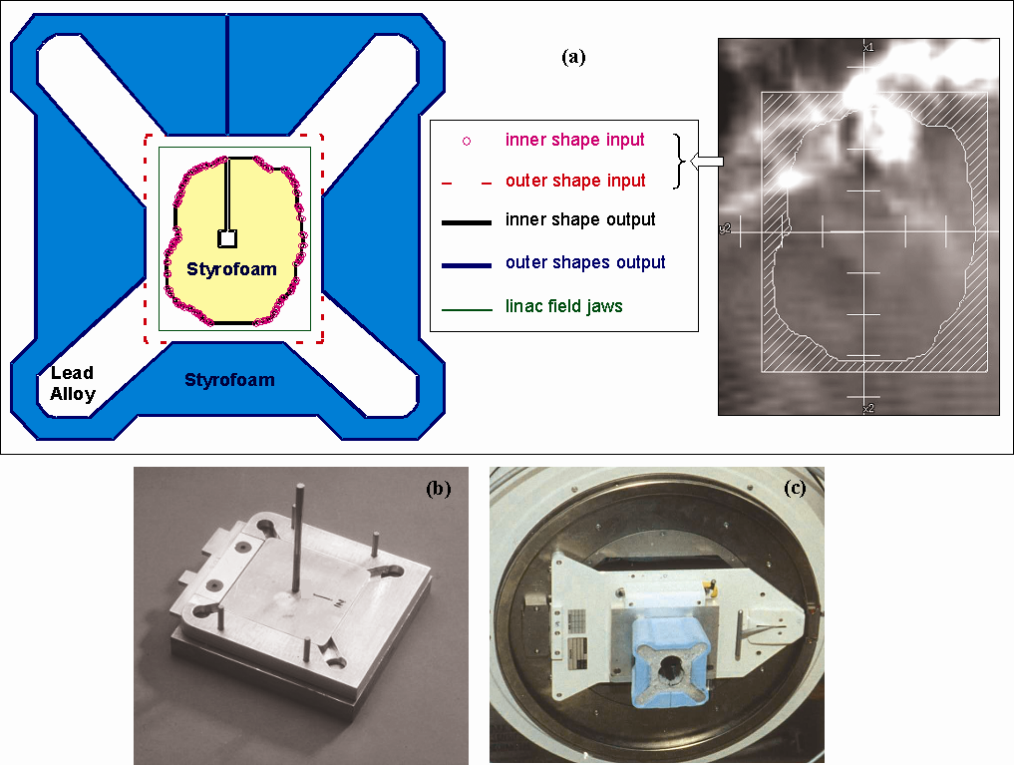
(a) The coordinates of a Pinnacle‐generated block (inset) are plotted together with the customization program output shapes giving the hot‐wire trajectories, which are exported to the block cutter. (b) A field‐coded base plate for locating the inner and outer Styrofoam shapes before pouring lead alloy. (c) A finished block attached to an Elekta SL15 LINAC.

(Figure 5a) shows the coordinates of a Pinnacle‐generated block plotted together with the program output shapes giving the hot‐wire trajectories for the block cutter. The original BEV is shown in the inset. The aperture and the outer block margin are reproduced while adding the central axis locating hole and outer fixing extensions. The inner and outer Styrofoam shapes are located on a field‐coded base‐plate before pouring lead alloy (Fig. 5(b). The inner Styrofoam piece is discarded after the lead has set. A finished block is depicted in Fig. 5(c).

The measured 50% isodose contours for the six irradiated blocks agreed with their corresponding BEVs to within 1 mm at the plane of the isocenter, with deviations of up to 1.5 mm over a maximum of 1 cm of the block perimeter. The light fields agreed with the 50% isodose contours to within 0.5 mm at the isocenter.

### D. Treatment verification

**Figure 6:**
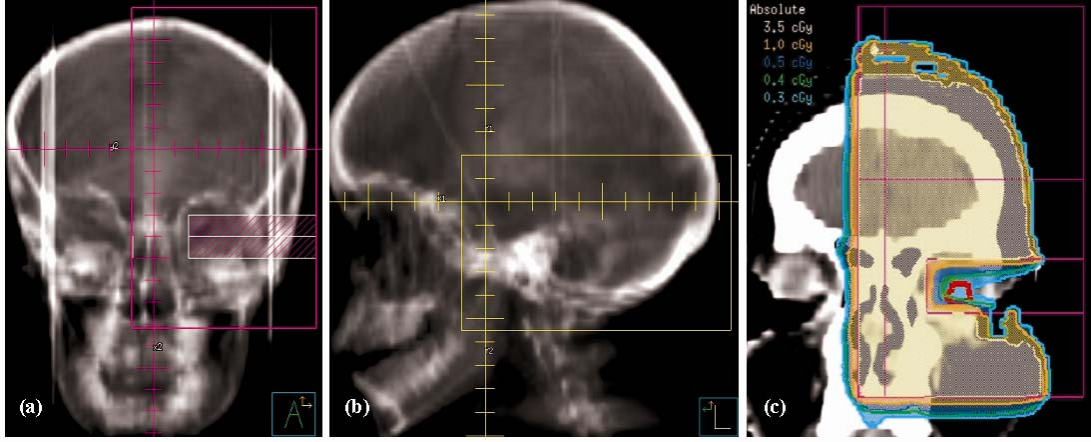
(a) Example AP isocenter verification DRR with MLC shielding, which is transferred to the EPI system. (b) The corresponding lateral verification field. (c) Coronal dose distribution through the left lens (red contour) for a 5‐MU irradiation by the AP beam (6 MV).

In the test patterns created in Pinnacle and imported into the EPI systems, the isocenter position was preserved to within one image pixel size (about 0.5 mm).

(Figure 6a) shows an AP verification field with MLC shielding to reduce lens dose while allowing extensive bony anatomy for image matching. The corresponding lateral verification field is shown in Fig. 6(b). A coronal dose distribution through the left lens for a 5‐MU, 6‐MV irradiation is presented in Fig. 6(c). The dose distribution was calculated by the beam model used in our general planning^(2)^ due to the limited field size with the SCRT model. Treatments of 20 children have been verified so far using fields with MLC shielding, reducing the lens dose by 12 to 15 times compared with the 5 cGy to 6 cGy delivered with non‐MLC fields. Typical mean and maximum computed lens doses were 0.4 cGy and 0.5 cGy, respectively. Pairs of thermoluminescent dosimeters (TLDs) placed on eyelids typically measured a dose of 0.5 cGy. Previous work on an anthropomorphic phantom showed a 10% overestimation of lens dose from AP beams by TLDs placed on eyelids.^(12)^ On that basis, the agreement between measured and computed doses is within 10% to 15%, which is acceptable for such small doses and so near the surface. On‐patient TLD measurements showed that the dose from treatment beams was less than 70 cGy over all 30 fractions, making the mean lens dose from the whole treatment less than approximately 75 cGy and 130 cGy with and without MLC shielding in AP verification beams, respectively, assuming portal images acquired on a maximum 10 fractions.

## IV. DISCUSSION AND CONCLUSIONS

As with the model for the Elekta SL15 LINAC, computed dose distributions with SCRT blocks for the Elekta Precise LINAC agreed with measurements well within published acceptability criteria.^(^ ^3^ ^–^ ^5^ ^)^ Concentrating on a limited set of treatment parameters (limited field size and depth ranges, single energy, one method of field shaping) has helped produce a model that is clinically acceptable for precise SCRT treatments. While sharing the same photon energy spectrum, the optimized SCRT models contained a different combination of parameters compared with those obtained using auto‐modeling with subsequent manual adjustments for general planning with field sizes 3 cm to 40 cm for wedged fields, but an identical parameter set for open fields. This is in contrast with our previous Elekta SL15 model,^(2)^ where the general‐planning and SCRT models included the same Monte Carlo‐generated energy spectrum but both were optimized manually from the start, which had led to the models being different for both cases of open and wedged fields. In addition, with the Monte Carlo‐plus‐manual optimization approach, four separate models had been necessary for the 3 cm to 40 cm field size range, whereas the auto‐modeling method required a single model. This could be a further benefit of auto‐modeling, since having multiple models may produce discontinuities and unexpected results in some cases. Since auto‐modeling is only applicable to unshielded fields within Pinnacle, it was not used for SCRT beam optimization, where the emphasis is predominantly on blocked fields. A model that satisfied the tight acceptability criteria of SCRT was achievable without the use of a Monte Carlo‐generated photon energy spectrum, as was used in the SCRT model for the first LINAC.^(2)^ This shows that the spectrum used does not necessarily have to be realistic.

Pinnacle does not force the computed 50% isodose contour to match the block shape at the isocenter plane. It models the energy fluence distribution exiting the head of the treatment unit as a 2D array with the area outside the aperture (formed by the block) removed from it. This array is used in the convolution and superposition steps. With our model, the resulting 50% isodose contour falls within 1.0 mm of the block edge at the isocenter plane with a 2×2×2 mm dose calculation grid. The better match shown by the digitized block (Fig. 2(d) could be due to that block having a slightly different shape since it was designed to match the measured 50% isodose. This may lead to dose grid effects since drawn contours are stored internally in Pinnacle at the resolution of the data set even if they are entered at a higher resolution. However, when a 1×1×1 mm dose grid was used, there was no discernible difference in the level of block‐border versus 50% isodose matching.

We routinely check the light field of each manufactured block against its BEV. Based on our measurements, we have set a tolerance of 1.0 mm for discrepancy in block edge position at the isocenter, with up to 1 cm of the perimeter allowed to differ by up to 1.5 mm. This keeps the block production accuracy within the 2.0 mm dose computation resolution normally used in clinical plans. Possible sources of error in block production include non‐parallelism between the hot wire and the beam central axis, nonvertical placements of the Styrofoam block into the block cutter, incorrect or variable wire temperature, and nonvertical central axis locator on the base plate (Fig. 5(b). The block export and customization software did not introduce any additional uncertainty in the planning process.

Dose prescription for SCRT at our center has been made for plans that have included density inhomogeneities derived from CT scans. Table C, including a combination of the RMI phantom manufacturer's data and additional measured densities for the CT QA one, was selected for clinical use since it demonstrated the closest agreement with measurements. Since the three tables are mainly different from each other at the higher densities and there is a limited amount of bone involved in cranial RT, the effect of relatively small differences in conversion tables results in dose calculation variations that are probably clinically insignificant. This is in agreement with other published work.^(^ ^6^ ^,^ ^13^ ^)^ In fact, it would be clinically acceptable to use any of the three tables for SCRT. The amounts of inhomogeneities present in the CT QA phantom make it a rather extreme test of the planning system's heterogeneity correction for most SCRT beams. However, this test highlights that using additional data points in the table, with densities ascertained for the actual inserts used in the experiment, reduces unnecessary systematic errors in calculated dose. Although the removal of such systematic discrepancies may not have a clinically significant impact on tumor or normal tissue dose, it is beneficial in reducing uncertainty margins for QA purposes (such as periodic testing of the planning system or independent checking of patients’ treatment plans) and as a matter of principle. The differences between the tables are expected to produce more pronounced variations for some extracranial treatments because (1) greater thicknesses of bone can be present in the path of a beam (e.g., femoral head, acetabulum and pubis), and (2) the largest improvement with the modified table is for average (noncortical) bone (Fig. 3). As demonstrated here, measured densities of the actual materials used can be different from manufacturers’ data by several percent. With Pinnacle and some other planning systems requiring the input of physical densities in contrast to electron densities, it is advisable to measure them where possible, as opposed to relying solely on published data. Manufacturers’ data should be treated as a guide, and measurement on the actual samples is recommended for accurate calculations.

Transfer of DRRs by the method described here has made treatment verification by EPI possible. Import of test patterns showed that the image transfer step did not introduce any errors into the verification process. The process, however, involves different steps and is time‐consuming. DICOM Derived Image Export Capability is included in version 6.2b of Pinnacle, which obviates the need for using FTP. However, it still requires image capture on Pinnacle and setting up of fields on the EPI system. The capability to export using the DICOM RT Image format, in which overlay information such as field dimensions, shielding, and isocenter position are exported together with the DRR image, would offer some time savings.

The use of MLC in AP isocenter verification fields reduces the lens dose by 12 to 15 times. However, even if the shielding is not used, the 5 cGy to 6 cGy lens dose per check for a phosphor‐screen EPI system does not pose a clinical problem. This is due to the treatment beams and the lateral check field all avoiding the lenses. Moreover, with our imaging protocol, isocenter verification is normally performed less than 10 times in the course of a 30‐fraction treatment. The total lens dose is usually well within the 5‐Gy cataract induction threshold for fractionated RT.^(14)^ We have also found that a satisfactory portal image can be obtained with 1‐MU irradiation using the iViewGT EPI system incorporating an amorphous silicon detector, offering a fivefold reduction in dose from check fields. Nevertheless, it is desirable to reduce dose to children's critical structures as low as reasonably achievable, especially since design, transfer, and delivery of MLC fields have become relatively efficient. MLC shielding may be more relevant to the verification of RT for infants^(15)^ or treatments that expose the lenses to a greater dose (e.g., whole brain RT).

The system described here has been in clinical use since 2001. It has provided a high level of precision as well as practical efficiency with the available resources. However, improvements are expected when planning system features such as DICOM RT Image export are implemented, as well as the provision of a micro‐MLC for treatment delivery to obviate the need for block production. The methodology described in this paper can also be applicable to other similar systems.

## ACKNOWLEDGMENTS

This work was funded in part by the Children's Cancer Unit Fund of The Royal Marsden NHS Trust. The authors would like to thank the following colleagues for their help: Ms. E. Adams, Dr. J. Bedford, Prof. M. Brada, Mrs. S. Helyer, Dr. R. Soomal, Mr. C. South and Mrs. H. Taylor.

## References

[1] Adams EJ , Suter BL , Warrington AP , Black B , Saran F , Brada M . Design and implementation of a system for treating pediatric patients with stereotactically‐guided conformal radiotherapy. Radiother Oncol 2001;60(3):289–297. 11514009 10.1016/s0167-8140(01)00383-8

[2] Bedford JL , Childs PJ , Hansen VN , Mosleh‐Shirazi MA , Verhaegen F . Warrington AP. Commissioning and quality assurance of the ADAC Pinnacle^3^ radiotherapy treatment planning system for external beam photons. Br J Radiol 2003;76(903):163–176. 12684232 10.1259/bjr/42085182

[3] Fraass B , Doppke K , Hunt M , et al. American Association of Physicists in Medicine Radiation Therapy Committee Task Group 53: *Quality assurance for clinical radiotherapy treatment planning.* Med Phys 1998;25(10):1773–1829. 9800687 10.1118/1.598373

[4] van Dyk J , Barnett RB , Cygler JE , Shragge PC . Commissioning and quality assurance of treatment planning computers. Int J Radiat Oncol Biol Phys 1993;26(2):261–273. 8491684 10.1016/0360-3016(93)90206-b

[5] Venselaar J , Welleweerd H , Mijnheer B . Tolerances for the accuracy of photon beam dose calculations of treatment planning systems. Radiother Oncol 2001;60(2):191–201. 11439214 10.1016/s0167-8140(01)00377-2

[6] Constantinou C , Harrington JC , DeWerd LA . An electron density calibration phantom for CT‐based treatment planning computers. Med Phys 1992;19(2):325–327. 1584125 10.1118/1.596862

[7] White DR , Martin RJ , Darlison R . Epoxy resin based tissue substitutes. Br J Radiol 1977;50(599):814–821. 588903 10.1259/0007-1285-50-599-814

[8] Barts and The London NHS Trust, London, UK Private communication 2003.

[9] White DR , Constantinou C , Martin RJ . Foamed epoxy resin‐based lung substitutes. Br J Radiol 1986;59(704):787–790. 3730777 10.1259/0007-1285-59-704-787

[10] International Commission on Radiation Units and Measurements . Tissue substitutes in radiation dosimetry and measurement. ICRU Report 44. 1989.

[11] Perks JR , Jalali R , Cosgrove VP , et al. Optimization of stereotactically‐guided conformal treatment planning of sellar and parasellar tumors, based on normal brain dose volume histograms. Int J Radiat Oncol Biol Phys 1999;45(2):507–513. 10487578 10.1016/s0360-3016(99)00156-x

[12] Reise SF , Donovan EM , Heisig S . Optimisation of dosemeter position for eye dose measurements. In: Proceedings of the Third Biennial Radiotherapy Physics Meeting of the Institution of Physics and Engineering in Medicine and Biology, Leeds, UK 1996 (Abstract).

[13] Guan H , Yin FF , Kim JH . Accuracy of inhomogeneity correction in photon radiotherapy from CT scans with different settings. Phys Med Biol 2002;47(17):N223–N231. 12361225 10.1088/0031-9155/47/17/402

[14] Henk JM , Whitelocke RA , Warrington AP , Bessell EM . Radiation dose to the lens and cataract formation. Int J Radiat Oncol Biol Phys 1993;25(5):815–820. 8478231 10.1016/0360-3016(93)90310-r

[15] Hall P , Granath F , Lundell M , Olsson K , Holm LE . Lenticular opacities in individuals exposed to ionizing radiation in infancy. Radiat Res 1999;152(2):190–195. 10409329

